# The burden of healthcare-associated infection in Ethiopia: a systematic review and meta-analysis

**DOI:** 10.1186/s41182-020-00263-2

**Published:** 2020-09-07

**Authors:** Abebaw Yeshambel Alemu, Aklilu Endalamaw, Wubet Alebachew Bayih

**Affiliations:** 1 Department of Paediatrics and Child Health Nursing, College of Health Sciences, Debre Tabor University, Debre Tabor, Ethiopia; 2 Department of Paediatrics and Child Health Nursing, School of Health Sciences, College of Medicine and Health Sciences, Bahir Dar University, Bahir Dar, Ethiopia

**Keywords:** Burden, Healthcare-associated infection, Meta-analysis, Ethiopia

## Abstract

**Background:**

Different primary studies in Ethiopia showed the burden of nosocomial infection across geographical setting and variant period. However, the national level of burden and types of healthcare-associated infections were unknown. Hence, this systematic review and meta-analysis estimated the overall nationwide burden and types of healthcare-associated infections in Ethiopia.

**Methods:**

We searched PubMed, Science Direct, Google Scholar, African Journal Online, and Addis Ababa University repository by date April 7, 2020. To assess publication bias, Egger’s test regression analysis was applied. Weight-inverse random-effect model meta-analysis was used. Subgroup analysis was conducted based on ward type, study region, study design, sample size and diagnostic method, ward type, and study participants.

**Results:**

A total of 18 studies with 13,821 patients participated in the overall prevalence estimation. The pooled prevalence of healthcare-associated infection was 16.96% (95% CI 14.10–19.82). Specifically, surgical site infection (39.66%), urinary tract infection (27.69%), bloodstream infection (19.9%), dual infections (SSI and UTI) (14.01%), and respiratory tract (13.51%) were the commonest types of healthcare-associated infection. In subgroup analysis, the highest overall prevalence was observed as surgical, gynecology, and obstetrics ward (22.42%).

**Conclusions:**

The national prevalence of healthcare-associated infection remains high. The most common type of HCAI was surgical site infection, followed by urinary tract infection, bloodstream infection, SSI and UTI, and respiratory tract infection. The overall prevalence was highest in surgical, gynecology, and obstetrics ward. Hence, infection prevention and control should be a priority agenda in healthcare with due emphasis for surgical patients.

## Background

Healthcare-associated infection (HCAI) is a preventable infection that a patient can encounter in a healthcare facility while receiving medical care [1, 2]. It occurs after 48 h of hospital admission, up to 3 days after discharge, or up to 30 days after the operation when someone was admitted for reasons other than infection [2]. Surgical site infection (SSI), urinary tract infection (UTI), bloodstream infection (BSI), and pneumonia or respiratory tract infection (RTI) were the commonest types of HCAI elsewhere [1].

According to the World Health Organization (WHO) 2019 HCAI fact sheet report, a hundred million patients were affected each year globally. This report added that the point prevalence of HCAI was estimated in the ranges between 3.5–12% and 5.7–19.1% in developed and low- and middle-income countries, respectively [3]. Nosocomial infection (NI) was found between 1.6 and 28.7% in Sub-Saharan Africa [4]. Specifically, the overall prevalence of HCAI was found in Botswana (13.4%) [5] and South Africa (8%) [6]. Regarding types of HCAI, a meta-analysis showed that RTI, UTI, SSI, and BSI from highest to lowest magnitude in order were found worldwide, but SSI (51.1%) was the leading in Africa [7]. Unlikely, nosocomial UTI was the utmost (35%) in Morocco Rabat hospitals [8].

Healthcare infections increase morbidity, mortality, long-term disability [9], hospital stay [10], microbial resistance to antibiotics [11], and healthcare costs for patients and their families [12]. Besides, it upsurges the financial burden on the healthcare system [9]. Hence, “clean care the safer care” program has been launched since 2004 with the WHO patient safety directive, which was aimed to reduce HCAI through improving hand hygiene practice at the center of achieving its aim [13]. Similarly, infection prevention and control guideline was developed for health facilities [14], and infection prevention and control policy recommendations initiated by WHO has been implemented in Ethiopia. Yet, HCAI remains the global public health problem elsewhere.

In the Ethiopian setting, different studies had been conducted to find the overall prevalence of HCAI and its type. The overall prevalence of HCAI was found in the ranges of 9 to 35.8% [15–18]. Concerning types of HCAI, SSI (13.5 to 52%) [15, 17, 18], RTI (5 to 19%) [12, 17], UTI (9.5 to 48%) [16, 18, 19], and BSI (4.3 to 46.6%) [20, 21] predominated in the country. However, all of the studies showed great variation across geographical setting and variant period. This discrepancy between studies makes it difficult to represent the national prevalence. Having national representative data is real to underpin effective prevention and control strategies. Thus, a need to have a pooled estimation of HCAI was necessitated at the country level. Therefore, this systematic review and meta-analysis aimed to estimate the overall pooled prevalence of HCAI and its types in Ethiopia.

## Methods and materials

### Reporting

The results of this review were reported based on the Preferred Reporting Items for Systematic Review and Meta-analysis (PRISMA) statement guideline [22] (Additional file 1 research checklist), and it is registered in the PROSPERO database:(PROSPERO ID CRD42020183158).

### Search strategy and information source

PubMed, Science Direct, Google Scholar, African Journal Online, and Addis Ababa University repository were searched. The core search terms and phrases were “prevalence”, “incidence”, “epidemiology”, “proportion”, “magnitude”, “burden”, “healthcare-associated infection”, “healthcare-acquired infection”, and “nosocomial infection”, “hospital acquired infection”, “surgical site infection”, “urinary tract infection”, “respiratory tract infection”, “pneumonia”, “ventilator associated pneumonia”, “bloodstream infection”, “central-line associated blood stream infection”, “skin and soft tissue infection”, and “Ethiopia”. The search strategies were developed using different Boolean operators. Notably, to fit advanced PubMed database, the following search strategy was applied on April 7, 2020: [(prevalence[MeSH Terms]) OR incidence[MeSH Terms]) OR proportion[MeSH Terms]) OR epidemiology[MeSH Terms]) OR magnitude[MeSH Terms]) OR burden[MeSH Terms]) AND healthcare-associated infection [MeSH Terms]) OR nosocomial infection [MeSH Terms]) OR hospital acquired infection[MeSH Terms]) OR healthcare-acquired infection[MeSH Terms]) OR healthcare infection[MeSH Terms]) OR surgical site infection[MeSH Terms]) OR urinary tract infection[MeSH Terms]) OR respiratory tract infection[MeSH Terms]) OR pneumonia[MeSH Terms]) OR ventilator associated pneumonia[MeSH Terms]) OR bloodstream infection[MeSH Terms]) OR central-line associated blood stream infection[MeSH Terms]) OR skin and soft tissue infection[MeSH Terms]) AND (Ethiopia)].

### Study selection

Retrieved studies were exported to the Endnote version 8 (Thomson Reuters, London) reference manager software to remove duplicate studies. Two independent reviewers screened the title and abstract. The disagreement was handled based on established article selection criteria. Two independent authors conducted the abstract and full-text review.

### Eligibility criteria

#### Inclusion criteria

Included studies were articles that reported the prevalence in general and/or at least one type of healthcare-associated infection. It also included studies published in English and studies conducted only in Ethiopia.

#### Exclusion criteria

Articles without full-text available and qualitative studies were excluded. Likewise, reviews, commentaries, consultants’ corners, letters, and conference abstracts were not considered.

### Quality assessment

We used Joanna Briggs Institute (JBI) quality appraisal criteria [23]. The tool consists of nine major items. The first item is appropriate to the sample frame. The second is the appropriate sampling technique. The third is the adequacy of the sample size. The fourth is a description of the study subjects and settings. The fifth is enough coverage of data analysis. The sixth is the validity of the method for identification of the condition. The seventh item is a standard and reliable measurement for all participants. The eighth is the appropriateness of statistical analysis. And the last item is adequacy and management of response rate. Studies were considered low-risk when it would fit 5 or above quality assessment checklists. Two independent authors appraised the quality of studies. The disagreement was resolved by the interference of a third reviewer.

### Data extraction

Two authors extracted data using the standardized format. The name of the first author and year, study region, study design, ward type, diagnostic method(s), sample size, the prevalence for overall, and types of HCAI were extracted. Whenever variations are observed, the phase was repeated. If discrepancies between data extractors continued, the third reviewer was involved.

### Outcome measurement

HCAI was considered, when reported as infection(s) acquired while receiving medical care based on culture confirmation [21, 24–27] or clinical and laboratory methods [10, 12, 15–20, 28–32]. Types of HCAI were any one or more of the following: SSI, UTI, BSI, RTI, or any other reported as healthcare-associated infection by included studies.

### Statistical analysis

The required data were collected using a Microsoft Excel 2013 workbook. Then, the STATA version 11 statistical software was used for meta-analysis. Publication bias was objectively checked using Egger’s regression test analysis [33]. Heterogeneity of studies was quantified using the I-squared statistic, in which 25, 50, and 75% represented low, moderate, and high heterogeneity, respectively [34]. Pooled analysis was conducted using a weighted inverse variance random-effects model [35]. Subgroup analysis was done by the region, study design, diagnostic method, and sample size. Besides, subgroup analysis was done based on ward type and study participants to overcome the inflation of the pooled effect from the inclusion of studies done across various wards and study participants with the age difference. Sensitivity analysis was employed to see the effect of a single study on the overall estimation.

## Results

### Literature search

**Fig. 1:**
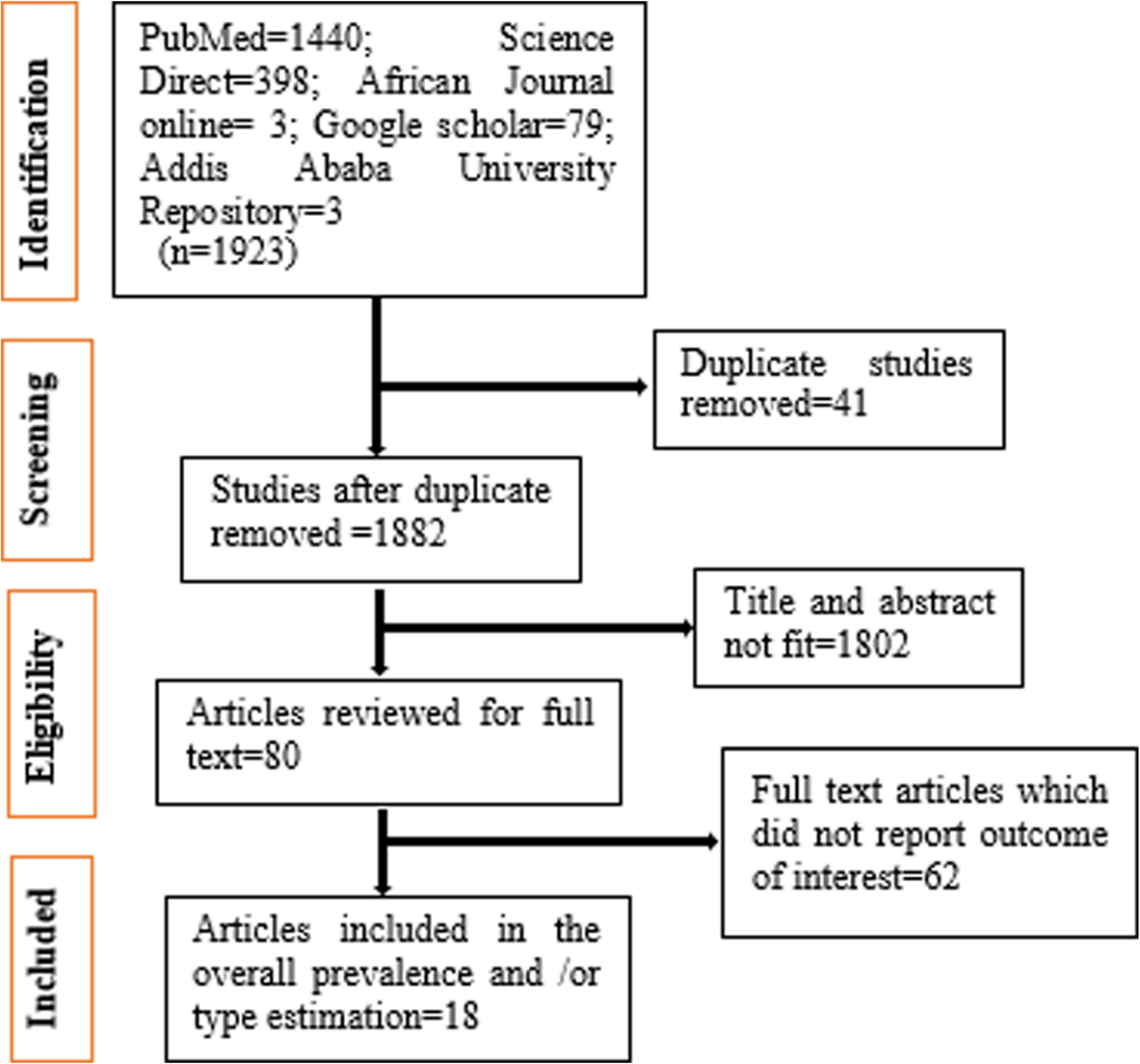
The study selection process

The search strategy retrieved 1440 articles from PubMed, 398 from Science Direct, 3 from African Journal Online, 79 from Google Scholar, and 3 from Addis Ababa University repository. After duplication was removed, 1882 remained. Eighty studies were screened for full-text review. Finally, 18 studies were used for meta-analysis (Fig. 1).

### Characteristics of included studies

Six studies were found in Addis Ababa [10, 12, 15, 27, 28, 32], five studies in Amhara region [16, 17, 24, 31, 36], five studies in Oromia [18–20, 25, 26], a study both in Addis Ababa and Southern Nations, Nationalities, and People’s Region (SNNPR) [29], one each in Tigray [30] and SNNPR [21]. Nine studies were done across all age groups. Eight studies were done on the adult population, and one study was done on pediatric patients. Fourteen studies used clinical and laboratory methods to diagnosis HCAI while the remaining were culture-confirmed. Four studies were conducted by using cohort study design and fourteen were cross-sectional. Only six studies included > 1000 sample size. The studies included in this systematic review and meta-analysis had no considerable risk. Therefore, all the studies were considered [10, 12, 15–21, 24–32] (Table 1).

**Table 1:** Characteristics and quality status of the studies in the meta-analysis of HCAI

First author year	Study region	Study design	Sample size	Prevalence	Quality status
Gedebou et al. 1987 [28]	Addis Ababa	Cross sectional	2506	13.40	Low risk
Gedebou et al. 1988 [10]	Addis Ababa	Cross sectional	700	17.00	Low risk
Habte-Gabr et al. 1988 [12]	Addis Ababa	Cohort	1006	16.40	Low risk
Berhe et al. 2001 [29]	Addis Ababa and SNNPR	Cohort	247	5.90	Low risk
Woldesenbet 2018 [27]	Addis Ababa	Cohort	435	8.5	Low risk
Tesfahun et al. 2009 [30]	Tigray region	Cross sectional	246	27.60	Low risk
Endalafer et al. 2011 [15]	Addis Ababa	Cross sectional	215	35.80	Low risk
Melaku et al. 2012 [16]	Amhara region	Cross sectional	1383	17.80	Low risk
Melaku et al. 2012 [31]	Amhara region	Cross sectional	1254	9.40	Low risk
Mulu et al. 2013 [24]	Amhara region	Cross sectional	294	10.90	Low risk
Sahile et al. 2016 [20]	Oromia region	Cross sectional	500	35	Low risk
Yallew et al. 2016 [17]	Amhara region	Cross sectional	908	14.90	Low risk
Tolera et al. 2018 [26]	Oromia region	Cross sectional	394	6.90	Low risk
Gashaw et al. 2018 [25]	Oromia region	Cross sectional	1015	11.60	Low risk
Ali et al. 2018 [18]	Oromia region	Cohort	1069	19.40	Low risk
Alemayehu et al. 2019 [21]	SNNPR	Cross sectional	939	21.40	Low risk
Gebremeskel et al. 2018 [32]	Addis Ababa	Cross sectional	410	19.80	Low risk
Zewdu et al. 2017 [19]	Oromia region	Cohort	300	14.00	Low risk

*SNNPR* Southern Nations, Nationalities, and People’s Region

Eleven of the studies have reported types of HCAI [10, 12, 15–18, 20, 21, 26, 28, 32] (Additional file 2). We assessed studies with JBI quality appraisal checklists. Based on this, none of the included studies was of poor quality status.

### Meta-analysis

#### The burden of healthcare-associated infection

The absence of publication bias was assessed with Egger’s regression test analysis (*p* = 0.456), which showed no publication bias.

**Fig. 2:**
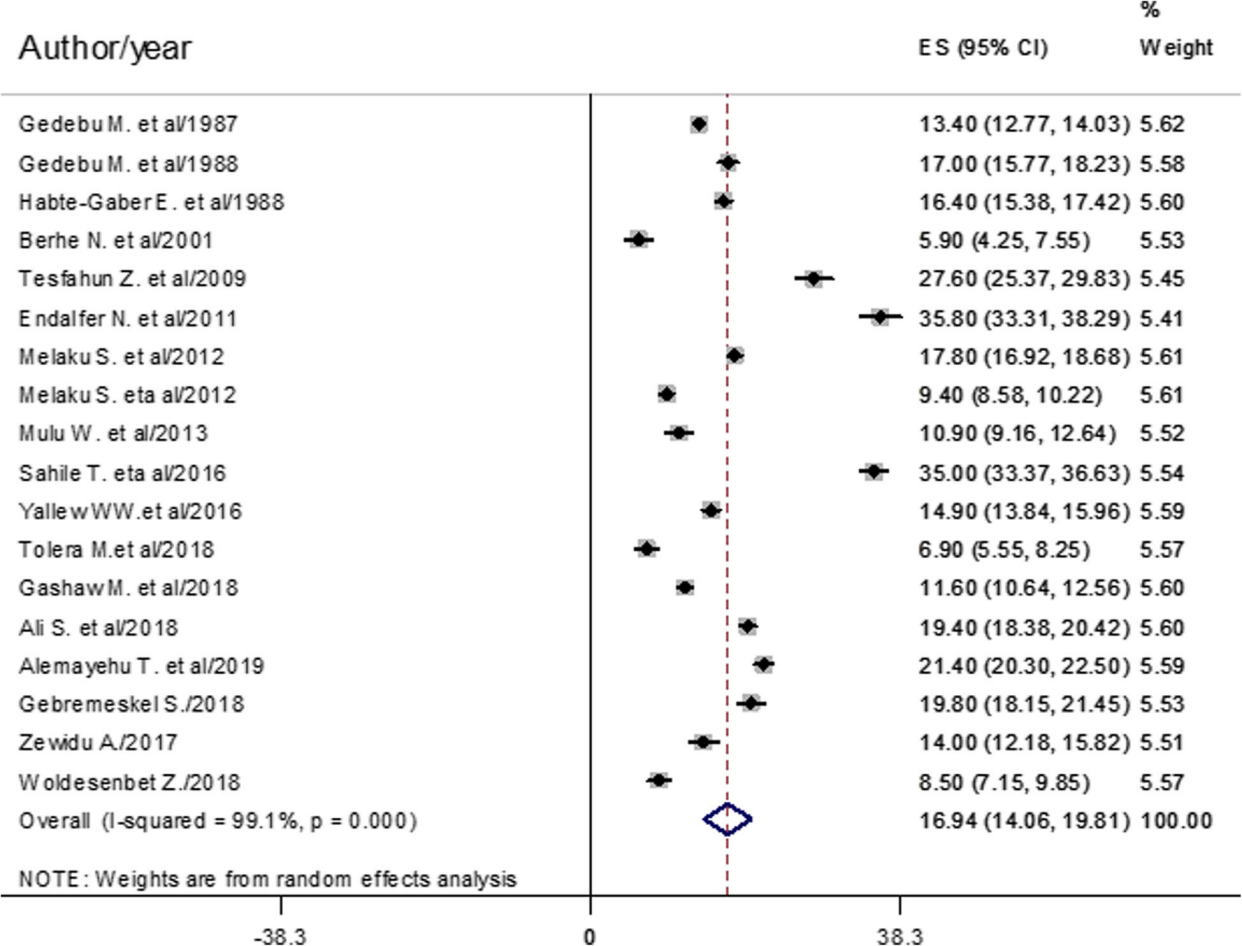
Forest plot of the overall pooled prevalence with corresponding 95% CIs of eighteen studies on HCAI

The pooled prevalence of healthcare-associated infection estimated from 18 studies [10, 12, 15–21, 24–32] was 16.94% (95% confidence interval (CI) 14.06–19.81) (Fig. 2).

**Fig. 3:**
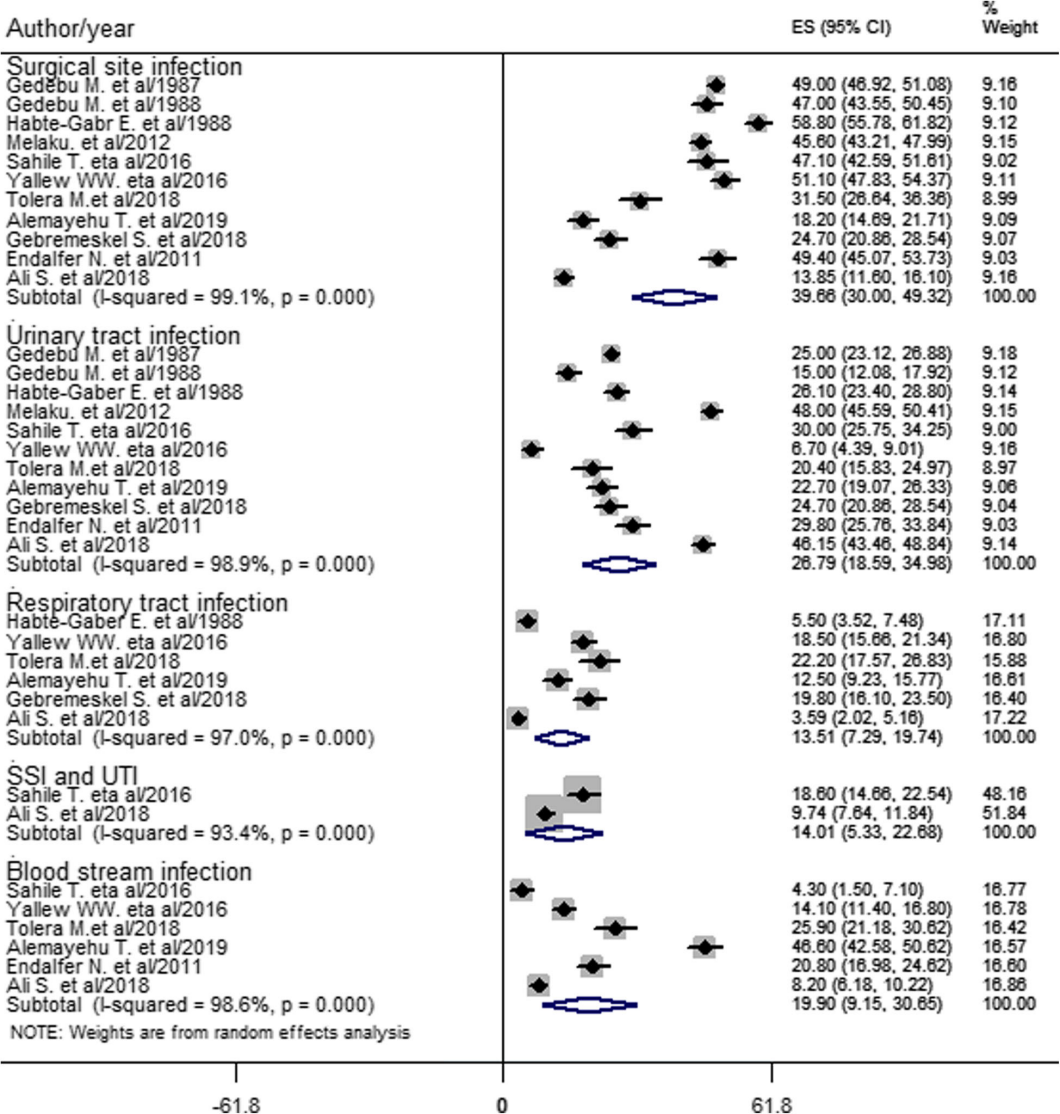
Forest plot of prevalence with corresponding 95% CIs of types of HCAI

Types of HCAI were estimated from the pooled effect of eleven studies [10, 12, 15–18, 20, 21, 26, 28, 32]. Accordingly, SSI (39.66%) was the most prevalent type of HCAI, followed by UTI (26.79%) and BSI (19.90%) estimated from six studies [15, 17, 18, 20, 21, 26]. Besides, six studies [12, 17, 18, 21, 26, 32] showed the prevalence of RTI (13.51%). Moreover, the dual infections of SSI and UTI estimated from two studies [18, 20] was 14.01% (Fig. 3).

#### Subgroup analysis

**Fig. 4:**
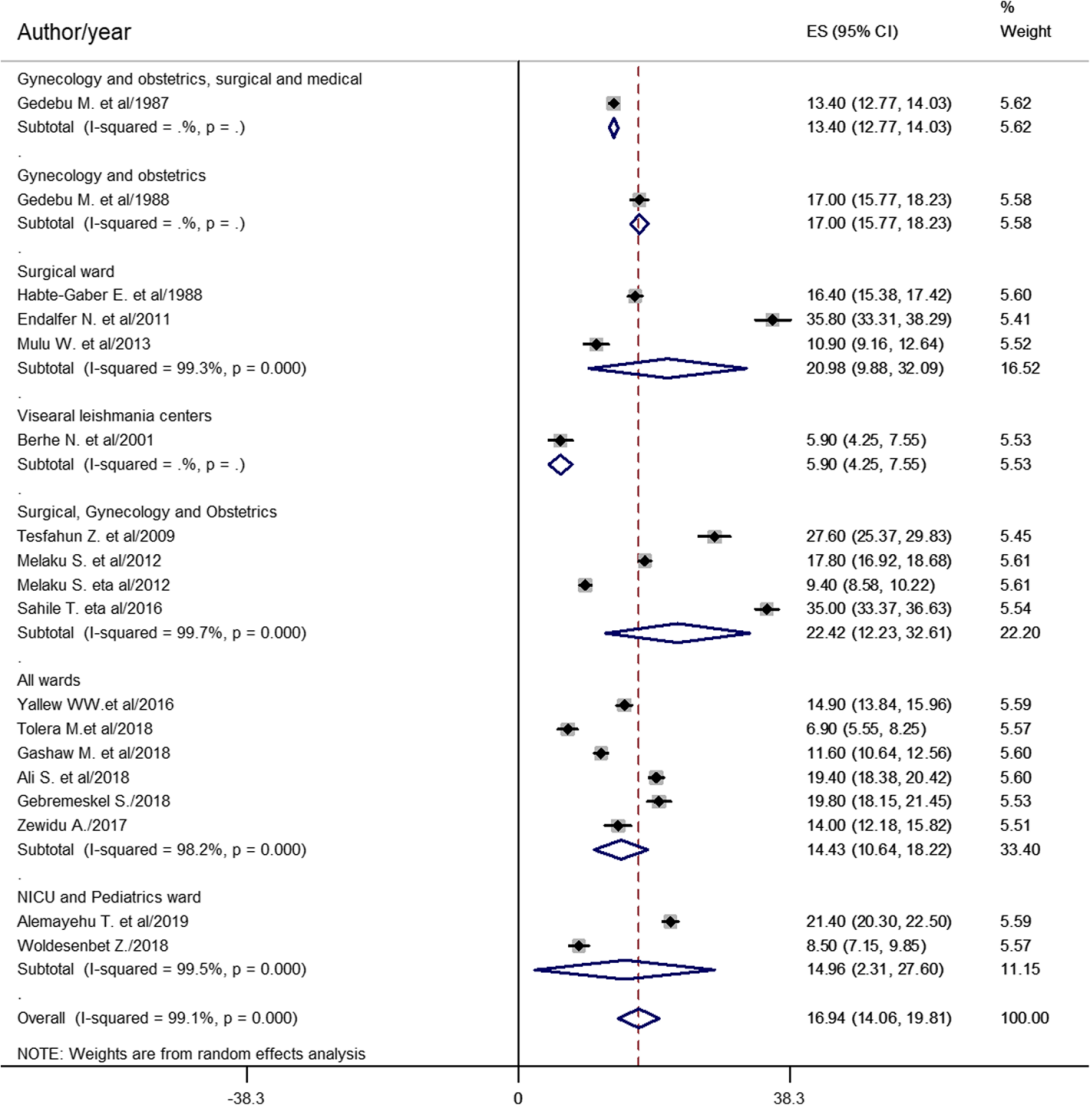
Forest plot of prevalence with corresponding 95% CIs of HCAI based on ward type

Subgroup analysis was done based on the study region, study design, diagnostic method and sample size, ward type, and study participant. Based on the pooled effect of two or more studies, HCAI estimated from six studies [10, 12, 15, 27, 28, 32] was found to be the highest in Addis Ababa (18.37%). Thirteen cross-sectional studies [10, 15–17, 20, 21, 24–26, 28, 30–32] found 18.52% HCAI rate in Ethiopia. Thirteen studies [10, 12, 15–20, 28–32] showed HCAI diagnosed by both clinical and laboratory methods was 18.89%. From the pooled effect of twelve studies [10, 15, 17, 19–21, 24, 26, 27, 29, 30, 32] using study sample < 1000, HCAI was 18.11% (Table 2). The subgroup analysis was also done based on ward type. The highest prevalence of HCAI estimated from four studies [16, 20, 30, 31] was 22.42% in surgical, gynecology, and obstetrics ward, followed by the surgical ward (20.98%) estimated from three studies [12, 15, 24] (Fig. 4).

**Table 2:** The prevalence of HCAI, 95% CI and heterogeneity estimate with a p-value for the subgroup analysis by study region, design, diagnostic method and sample size

Variables	Characteristics	Pooled prevalence (95% CI)	***I***^**2**^ (***p*** value)
By region	Addis Ababa	18.37% (13.91–22.82)	98.8% (0.000)
	Oromia	17.37% (9.19–25.56)	98.5% (0.000)
	Amhara	13.27% (9.00–17.52)	99.5% (0.000)
	Tigray	27.6% (25.37–29.83	-
	SNNPR	21.4% (20.3–22.5)	-
	Addis Ababa and SNNPR	5.9% (4.25–7.55)	-
Study design	Cross sectional	18.52% (14.91–22.12)	99.3% (0.000)
	Cohort	12.86% (7.98–17.74)	98.4% (0.000)
Diagnostic method	Clinical and laboratory	18.89% (14.85–21.51)	99.2% (0.000)
	Culture confirmed	12.71% (6.4–19.02)	99% (0.000)
Sample size	< 1000	18.11% (13.18–23.04)	98.6% (0.000)
	≥ 1000	14.66% (11.72–17.60)	99.3% (0.000)

*SNNPR* Southern Nations, Nationalities, and People’s Region

**Fig. 5:**
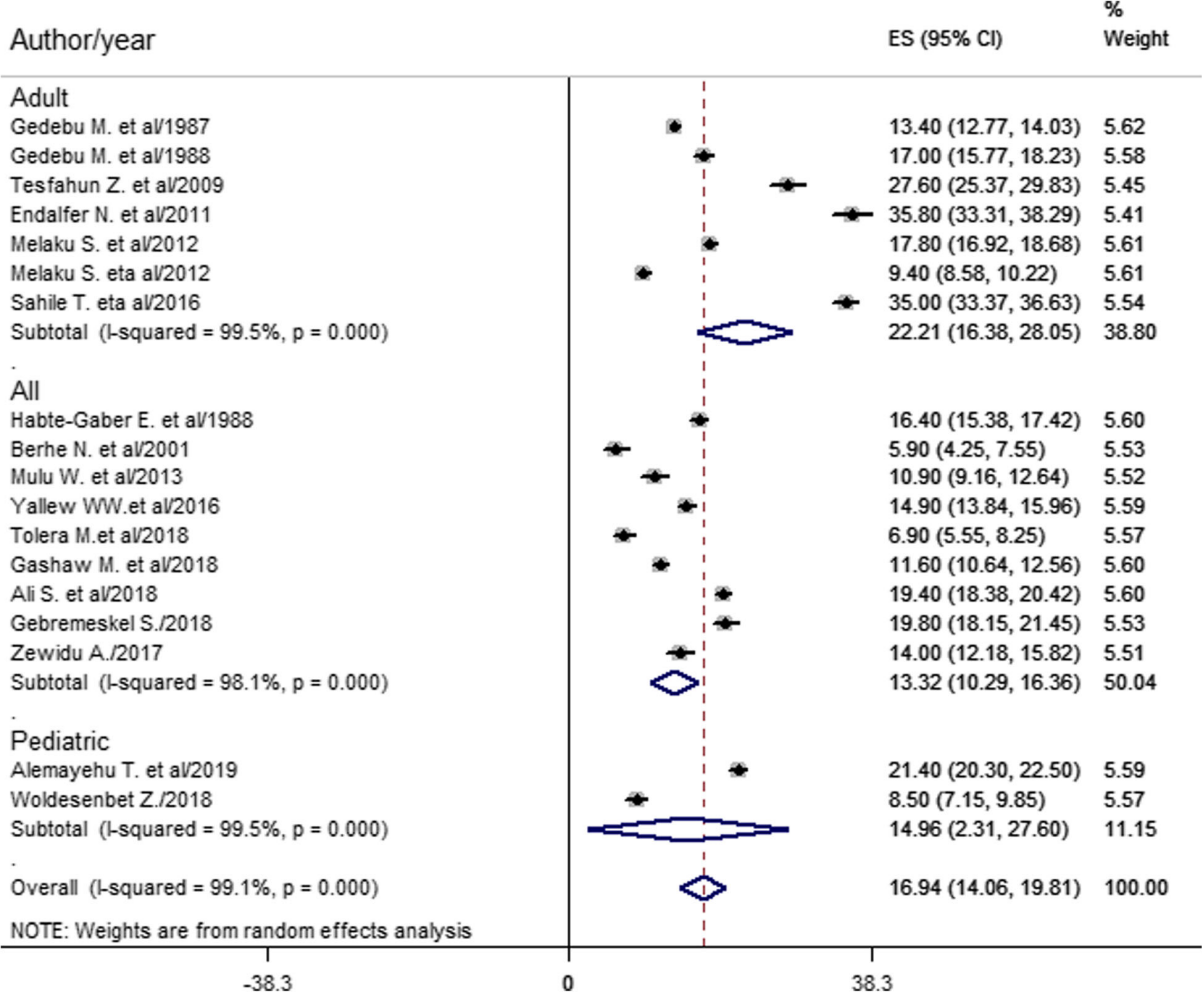
Forest plot of prevalence with corresponding 95% CIs of HCAI based on study participants

Based on the subgroup analysis of study participants, the pooled prevalence of HCAI estimated from seven studies [10, 15, 16, 20, 28, 30, 31] was 22.21% among the adult population (Fig. 5).

#### Sensitivity analysis

**Fig. 6:**
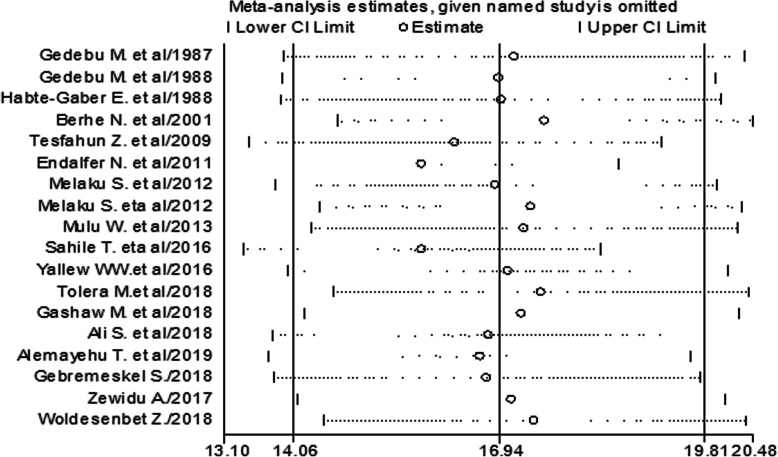
The overall pooled prevalence sensitivity analysis of HCAI when the studies omitted step by step

Endalafer et al. [15] and Sahile et al. [20] had shown an impact on the overall estimation of the meta-analysis result (Fig. 6).

## Discussion

The Sustainable Development Goal (SDG) goal-3 is implemented to ensure access to quality essential healthcare services in every country [37]. However, HCAI confronts quality in healthcare systems that fail to build strong principles to reduce the risks and spread of nosocomial infection [38]. Thus, HCAI has been continued as preventable causes of morbidity, mortality, and spending health system cost.

This meta-analysis estimated the country level overall pooled prevalence of healthcare-associated infection and types in Ethiopia. Accordingly, the overall pooled prevalence of HCAI was 16.94% (95% CI 14.06–19.81) (Fig. 2). This result was higher than studies done in China (3.12%) [39], Morocco (10.3%) [8], Botswana (13.54%) [5], and South Africa (7.67%) [6]. The possible reasons for high prevalence in this study might be very low hand hygiene practice, low adherence to infection prevention practice, low level of job satisfaction, morally distressed nurses, resource constraints, low implementation of the nursing process, and less attention given to healthcare-associated infection.

Keeping hand hygiene before patient contact is vital to prevent NI. However, only 7% of physicians working at two university hospitals in Addis Ababa Ethiopia performed hand hygiene before patient contact [40]. Evidence also showed that 35% of nurses were non-adherent to infection prevention practices in Southwest Ethiopia [41]. Besides, nearly 68% of health professionals were less satisfied with their work in the Amhara region [42]. Additionally, about 84% of nurses were morally distressed in the northwestern part of the country [43]. As a result, acquisition of HCAI from unsafe hands of unsatisfied healthcare providers augmented by less adherence to infection prevention and morally less prepared nurses might limit the quality of patient care in healthcare facilities. These may in turn increase HCAI in the country.

Healthcare workers (HCWs) disposed of that lack of hand hygiene agents and sinks in public hospitals of Addis Ababa Ethiopia [40]. The implementation of the nursing process was below half (49%) in the northwestern part of the country [44]. Furthermore, healthcare providers, patients, and/or families are more curious about the primary reason for admission or healthcare visits. Therefore, resource shortage preclude infection prevention practice, patients at risk of HCAI followed without the nursing process would not get nursing intervention, and less attention given for HCAI could justify high prevalence.

This systematic review and meta-analysis found that the most prevalent HCAI is SSI. This is greater than studies done in Morocco [8], Botswana [5], South Africa [6], and a meta-analysis done in mainland China [39]. The higher prevalence in the current finding might be due to poor surgical instrument processing, inadequate knowledge of healthcare workers (HCWs) about SSI prevention, the highest occurrence of HCAI in the surgery ward, poor SSI prevention practice by HCWs, and surgical procedure errors in Ethiopian settings. None of the HCWs in Addis Ababa Ethiopia were using biological indicators of steam (*Bacillus stearothermophilus*) or dry (*Bacillus subtilis)* heat sterilization [45]. Knowledge of SSI prevention was found inadequate, and SSI prevention was practiced poorly at two referral hospitals in northwest Ethiopia [46]. Besides, as shown in Fig. 4 the prevalence of HCAI was the second highest in the surgery ward (20.98%). Regarding surgical procedure errors, surgical and medical error claims in the country showed that medical error was 26.7%, and the majority (72%) of the complaints were surgical related and emerged from the operation room [47]. As a result of the above antecedents, prevalence of nosocomial SSI could be higher in the country.

A higher prevalence of SSI and an abundance of NI in the surgery ward reported in the current meta-analysis might be explained by the low density of healthcare providers in Ethiopia. The density of physicians (per 1000 population) was 0.0, 0.6, and 0.8 in Ethiopia, Morocco, and South Africa respectively while the density of nursing and midwifery personals (per 1000 population) was 0.3, 0.9, and 5.2 in Ethiopia, Morocco, and South Africa orderly [48]. Nurses, midwives, and physicians serving many people every day might be unable to maintain healthcare quality, so this might be another reason for higher HCAI in the surgery ward. On top of the abovementioned reasons, nurse’s burnout might contribute to the high prevalence of SSI and an abundance of HCAI in the surgery ward. In the United States (US) nurse’s burnout was found as a single most important associated factor for increased nosocomial UTI and SSI [49].

## Strength and limitation

This systematic review and meta-analysis was the first national estimation conducted in Ethiopia. However, it may lack national representativeness because no data were found from Benishangul Gumuz, Afar, Gambella, Somalia, Dire Dawa, and Harare regions of Ethiopia.

## Conclusions

In this finding, the burden of HCAI remains high. SSI was the first most common type of HCAI, followed by UTI, BSI, SSI and UTI, and RTI from highest to lowest magnitude respectively. The highest overall burden was observed in surgery, gynecology, and obstetrics ward followed by surgical ward. Hence, infection prevention and control should be strengthened and held as the priority agenda in healthcare with due emphasis for surgical site infection.

## Supplementary information

**Additional file 1.** PRISMA 2009 Checklist.

**Additional file 2.** Studies to estimate HCAI.

## Data Availability

All data generated or analyzed during this study are included in this published article.

## Abbreviations

AOR: Adjusted odds ratio
BSI: Blood stream infection
CI: Confidence interval
HCAI: Healthcare-associated infections
HCWs: Healthcare workers
LMIC: Low- and middle-income country
NI: Nosocomial infection
RTI: Respiratory infection
SNNPR: Southern Nations, Nationalities, and People’s Region
SDG: Sustainable Development Goal
SSTI: Skin and soft tissue infection
UTI: Urinary tract infection
WHO: World Health Organization
