# Supplementary material for: The burden of healthcare-associated infection in Ethiopia: a systematic review and meta-analysis
Source: Trop Med Health. 2020 Sep 7;48:77. doi: 10.1186/s41182-020-00263-2 (PMC7487565; doi:10.1186/s41182-020-00263-2)
Supplement: Supplementary file 2 — Additional file 2. Studies to estimate HCAI. [file 41182_2020_263_MOESM2_ESM.docx]

| **First author year** | **Region** | **Study design** | **Sample size** | **HCAI type** | **Prevalence** | **Ward type** |
| --- | --- | --- | --- | --- | --- | --- |
| Gedebu M. et al/1987 [28] | Addis Ababa | Cross sectional | 336 | SSI | 49% | Gynecology and obstetrics, surgical and medical |
|  |  |  |  | UTI | 25% |  |
| Gedebu M. et al/1988 [10] | Addis Ababa | Cross sectional | 119 | SSI | 47% | Gynecology and obstetrics |
|  |  |  |  | UTI | 15% |  |
| Habte-Gaber E. et al/1988 [12] | Addis Ababa | Cohort | 165 | SSI | 58.8% | Surgical ward |
|  |  |  |  | UTI | 26.1% |  |
|  |  |  |  | RTI | 5.5% |  |
| Endalfer N. et al/2011 [15] | Addis Ababa | Cross sectional | 77 | SSI | 49.4% | Surgical ward |
|  |  |  |  | UTI | 29.8% |  |
|  |  |  |  | BSI | 20.8% |  |
| Melaku S. et al/2012 [16] | Amhara region | Cross sectional | 246 | SSI | 45.6% | Surgical, Gynecology and Obstetrics |
|  |  |  |  | UTI | 48% |  |
| Sahile T. eta al/2016 [20] | Oromia region | Cross sectional | 70 | SSI | 47.1% | Surgical, Gynecology and Obstetrics |
|  |  |  |  | UTI | 30% |  |
|  |  |  |  | SSI and UTI | 18.6% |  |
|  |  |  |  | BSI | 4.3% |  |
| Yallew WW.et al/2016 [17] | Amhara region | Cross sectional | 135 | SSI | 51.1% | All wards |
|  |  |  |  | UTI | 6.7% |  |
|  |  |  |  | BSI | 14.1% |  |
|  |  |  |  | RTI | 18.5% |  |
| Tolera M.et al/2018 [26] | Oromia region | Cross sectional | 54 | SSI | 31.5% | All wards |
|  |  |  |  | UTI | 20.4% |  |
|  |  |  |  | BSI | 25.9% |  |
|  |  |  |  | RTI | 22.2% |  |
| Ali S. et al/2018 [18] | Oromia region | Cohort | 195 | SSI | 13.85% | All wards |
|  |  |  |  | UTI | 46.15% |  |
|  |  |  |  | BSI | 8.2% |  |
|  |  |  |  | RTI | 3.59% |  |
|  |  |  |  | SSI and BSI | 9.74% |  |
| Alemayehu T. et al/2019 [21] | SNNPR | Cross sectional | 88 | SSI | 18.2% | All wards |
|  |  |  |  | UTI | 22.7% |  |
|  |  |  |  | BSI | 46.6% |  |
|  |  |  |  | RTI | 12.5% |  |
| Gebremeskel S. et al/2018 [32] | Addis Ababa | Cross sectional | 81 | SSI | 24.7% | All wards |
|  |  |  |  | UTI | 24.7% |  |
|  |  |  |  | BSI | 19.8% |  |

Note: SSI: Surgical Site Infection; BSI: Blood Stream infection; UTI: Urinary Tract Infection; RTI: Respiratory Tract Infection; SNNPR: Southern Nations Nationalities and Peoples Region;
